# Authoritative parenting stimulates academic achievement, also partly via self-efficacy and intention towards getting good grades

**DOI:** 10.1371/journal.pone.0265595

**Published:** 2022-03-30

**Authors:** Joyce Hayek, Francine Schneider, Nathalie Lahoud, Maya Tueni, Hein de Vries

**Affiliations:** 1 School for Public Health and Primary Care (CAPHRI), Department of Health Promotion, Maastricht University, Maastricht, The Netherlands; 2 Department of Nutrition and Dietetics, Faculty of Sciences II, Lebanese University, Fanar, Lebanon; 3 National Institute of Public Health, Clinical Epidemiology & Toxicology (INSPECT-LB), Beirut, Lebanon; 4 Faculty of Public Health, Lebanese University, Fanar, Lebanon; Sam Houston State University, UNITED STATES

## Abstract

**Background:**

The aim of this prospective study is to examine how parenting style relates to academic achievement of Lebanese adolescents and test the mediating effect of self-efficacy and intention towards getting good grades. Potential moderation by demographic factors (age, gender, school type, religion and parents’ education) was also examined.

**Methods:**

Students (n = 345) from private and public schools in Mount Lebanon and Beirut area, aged between 15 and 18, participated in a two-wave longitudinal study and completed a self-administered questionnaire based on the I-Change Model assessing socio-demographics (age, gender, school type, parents’ education, family structure, religion), socio-cognitive factors (attitude, social norms, self-efficacy, intention), parenting styles and academic achievement. Adolescent were surveyed at two time points, six months apart. A multiple linear regression was carried out to identify baseline factors independently associated with academic achievement 6 months later. Moderation was examined using Hayes’s SPSS macro PROCESS. A serial mediation model was employed to test for the sequential mediating effect of self-efficacy and intention between parenting style and academic achievement.

**Results:**

Authoritative parenting was prospectively associated with better academic achievement and higher self-efficacy and intention at 6 months follow up. In addition, self-efficacy and intention towards getting good grades were found to mediate the relationship of parenting style to academic achievement. Adolescents who perceive their parents as authoritative are more likely to develop high efficacy beliefs and higher intention and subsequently are more likely to achieve better in school compared to peers of neglectful parents. Socio-demographics did not moderate the effect of parenting on academic achievement.

**Conclusion:**

Authoritative parenting influenced both directly and indirectly the academic achievement of their children. Interventions aiming at improving academic performance of adolescents should also encompass positive parenting style strategies.

## Introduction

Good academic achievement increases the chances for a successful future for both individuals and societies [1, 2]. Addressing key factors that influence academic performance would help in creating better opportunities for youth and securing a better future in terms of better health and higher quality of life [3]. According to Bronfenbrenner ecological model, adolescents’ behaviors and outcomes are influenced by various levels of environment [4]. The microenvironment is the most immediate environment in which adolescents live, such as the school or home environment, and is considered to have the strongest impact on adolescent development [5]. Parents who are part of the home environment may have a direct and indirect influence on their children’s outcomes via their parenting styles [6]. Parenting style is the emotional context in which parents’ behaviors are expressed in the effort to socialize their child [7]. Four parenting styles can be defined based on the combination of two dimensions of parenting behavior—demandingness and responsiveness: authoritative parenting (high on demandingness and responsiveness), authoritarian (high on demandingness and low on responsiveness), permissive (high on responsiveness and low on demandingness) and neglectful (low on demandingness and responsiveness) [8, 9].

### Parenting style influence on academic achievement and the role of social cognitions-literature review

Parenting styles have been found to significantly influence several child outcomes among which eating behaviors [10], substance use [11], psychological outcome [12] and educational outcome [13]. In regard to academic performance, and while findings may vary across cultures and social groups [14, 15], authoritative parenting has been generally found to have the most positive outcomes and promote higher academic achievement [16–18] while neglectful parenting has been consistently linked with the poorest outcomes and lower grades [19]. Evidence also exists on the indirect effect of parenting on adolescent’s achievement [20, 21] pointing to a dual influence of parents both direct and indirect. The family environment is assumed to influence behavior both directly as well as indirectly through mediating variables such as cognitive mediators reflecting a dual process view [22–24]. The dual process suggests that environmental factors, in this case parents, can have a direct automatic influence on behavior and an indirect mediated effect via behavior-specific cognition [24]. According to social cognitions theories, attitude, subjective norm and perceived self-efficacy are the central cognitions that are believed to influence behavioral intention which is considered the primary determinant of behavior [25, 26]. Intention reflects the motivation and intent to perform a given behavior and is determined by three constructs; Attitude refers to the perceived pros and cons of a certain behavior, social norms indicates the perceived social pressure to engage or not in a given behavior and self-efficacy refers to the belief in one’s capabilities to execute specific behaviors to produce positive outcomes [25, 27]. The more favorable the attitude, perceived social norms and efficacy beliefs, the strongest the intention to engage in the behavior in question [25]. Another construct that is believed to influence intentional behavior is motivation [28]. Motivation explains why individuals decide to do something, how hard they would work to achieve it and how long they would persevere to sustain it [29]. According to the Self-Determination Theory, motivation which can be intrinsic (emerging from personal interest) or extrinsic (prompted by external force and social values) can determine the strength of intentional behavior [29, 30]. Autonomous intentions that are driven by intrinsic motivation are more likely to be translated into behavior and more likely to be sustained than controlled intentions [28]. Parenting style can impact academic performance through its influence on social-cognitive factors. For instance, a certain parenting style may promote positive attitudes toward education or a higher academic self-efficacy which will influence the intention to obtain good grades and subsequently impact academic outcome. Indeed, many studies have demonstrated that parenting styles can help foster the development of healthy psychosocial competencies which in turn affect scholastic performance [17, 31, 32]. Adolescents from authoritative parents were found to have higher self-efficacy beliefs compared to adolescents from authoritarian and permissive parents [18, 33]. Adolescents’ achievement beliefs can influence their achievement-related behaviors [34], adolescent with high beliefs are more motivated, set higher challenging academic goals, put more effort working towards those goals and are more resilient in the face of difficulties and subsequently perform higher [35–37].

### Gap-building on previous research

Several studies describe the relation of parenting on academic outcome of their offspring [15, 17, 38, 39], including the role of socio-cognitive factors on academic performance [40, 41]. Even though there are many studies on the dual influence of the environment on general behaviors such as health behaviors [42], research examining the dual effect of parenting, the direct and indirect effect through the mediating effect of cognitive factors on academic performance is limited. Hence, our study aims to further elucidate the mechanism by which parenting influence adolescent’s achievement using an integrated approach. Gaining insight into the direct relation of parenting to academic achievement and how cognitions may mediate this relation is highly relevant in order to inform targeted intervention development. In addition, there is a need to study the influence of parenting styles in different cultures such as the MENA region and specifically in the Lebanese context where empirical evidence of this kind is lacking. The latter will add to the existing literature and expand our knowledge on parenting socialization in different cultural context. On the other hand, student performance in Lebanon, as measured by international assessments such as the Programme for International Student Assessment (PISA) has been shown to be significantly lower than other countries and point to a learning gap [43]. Lebanese were on average three to four years of school behind peers from other countries. Results also showed that 60% of Lebanese students did not achieve basic proficiency in math, 62% in science and 68% in reading, placing them at high risk of exclusion from secondary school [43]. Understanding how parenting style may influence academic achievement of Lebanese adolescents will help practitioners and policymakers understand how to capacitate Lebanese adolescent reach their full academic potential.

### Study objectives

Hence, the first objective of this study is to examine if parenting style prospectively influences academic performance of Lebanese adolescents. The second objective is to explore which social-cognitive factor mediates the influence of parenting on academic achievement. In addition, since it has been postulated that parenting may have a differential impact on adolescent outcome depending on socio-demographic characteristics [44], the third objective is to investigate whether adolescent age, gender, religion, the type of school they are enrolled in as well as their parent’s education might moderate the influence of parenting style on academic achievement of Lebanese adolescents.

## Materials and methods

### Design

This prospective study was based on a secondary analysis of data from a larger three-wave longitudinal research project investigating the predictors of academic achievement in Lebanese adolescents [45]. As part of this project, adolescents aged 15 to 18 from private and public schools completed a survey assessing socio-demographics, lifestyle factors and motivational factors. The students were surveyed at three-point time: time 1 (t1), after six months (t2) and after 12 months (t3). For the present study data from the second (t2) and third wave (t3) of the survey were utilized as parenting style was not assessed at baseline. The predictor variable parenting style was taken at t2, the control variables socio-demographics and the potential mediators socio-cognitive variables were also taken at t2 and the outcome variable academic achievement was taken at t3.

### Participants

The baseline sample was a total of 600 adolescents out of which 563 (94%) with valid data, aged 15 to 18, from public and private schools across Beirut and Mount Lebanon area. Participating schools were randomly selected form the Ministry of Education’s list. The directors of these schools were approached face-to-face and provided with the study questionnaire and seven (four private and three public) agreed to partake in the study. From these schools, all students enrolled in grade 10 and 11 were invited to participate in the survey. For the current study only participants with complete data set at t2 and t3 were included resulting in a total sample of 345 adolescents (61.27%).

### Procedure

After obtaining the school director’s consent to conduct the study, trained dietitians visited the schools that agreed to participate and distributed pen-paper survey to all students. The trained dietitians were present at all time in the classroom and read aloud each question with the corresponding answers and were available for any help or clarification. The survey took approximately one hour to be completed. The study questionnaire was reviewed and approved by the Lebanese Ministry of Education and Higher Education and the study was conducted in accordance with the Declaration of Helsinki [46]. Written informed consent was obtained from all students and their parents prior to participating in the study and ethical approval was obtained from Al Hayat Hospital ethical committee.

### Measurements

#### Demographic factors (t2)

Socio-demographic variables included students’ sex (1 = male; 2 = female), age (1 = 15; 2 = 16; 3 = 17; 4 = 18), type of school (1 = public; 2 = private), educational level of parents (1 = low: never went to school & primary school; 2 = medium: complementary & secondary school; 3 = high: technical school & university), family structure (1 = living with both parents; 2 = other arrangements) and religion (0 = Non-Christian, 1 = Christian).

#### Parenting style (t2)

The Authoritative Parenting Index (API) was used to assess parenting styles [47]. The API measures the responsive and demanding dimensions of parenting behavior as perceived by the adolescents. Nine items measured responsiveness (e.g. “She/he listens to what I have to say”) and seven items measured demandingness (e.g.” She/he has rules that I must follow”). In this study the items were presented once in reference for both parents (e.g. “They make sure I go to bed on time”). Students used a four-point response scale (1 = Not like them, 2 = Sort of like them, 3 = A lot like them and 4 = Just like them) to indicate how well the statements describe their parents’ parenting styles with higher scores indicating higher levels of responsiveness and demandingness. The latter yielded two separate scores for each participant, namely responsiveness and demandingness. The final scale scores could range from 9–36 for responsiveness (Cronbach’s α = 0.80) and 7–28 for demandingness (Cronbach’s α = 0.70). Parenting styles were created using median splits on demandingness and responsiveness. A parent who was rated high on both responsiveness and demandingness (above the median 30 for responsiveness, 16 for demandingness) was categorized as authoritative (N = 107). A parent who was rated low on both responsiveness and demandingness (below the median) was categorized as neglectful (N = 66). A parent who was rated high in responsiveness and low in demandingness was categorized as permissive (N = 68). A parent rated as low on responsiveness and low in demandingness was categorized as authoritarian (N = 100).

#### Socio-cognitive factors (t2)

The I-Change model [26] was used as a framework for including socio-cognitive variables as determinants and potential mediators for academic achievement. Attitude was measured with four questions assessing the pros and cons of getting good grades. Two questions measured positive attitudes “Getting good academic grades is a good help for getting a good job/will get me compliment from my parents”. The responses were coded from– 2 (strongly disagree) to +2 (strongly agree). Two questions measured negative attitudes “Getting good academic grades means that I have to work too hard/will cause disapproval among my friends” using the same scale reverse coded from +2 (strongly agree) to -2 (strongly disagree) so that higher scores reflect a more positive attitude towards getting good grade.

Social norms were measured using the responses to three questions asking if parents or teacher expect adolescents to get good academic grades “My father/my mother/my teacher expects me to get good academic grades” on a five-point Likert scale ranging from +2 (strongly agree) to -2 (strongly disagree).

Self-efficacy was assessed using responses to five questions “I find it easy to get good academic grades/to concentrate at school for getting good academic grades/to master the skills that are taught in class this year/to concentrate on school work when I am at home/to finish all my school work” on a scale ranging from +2 (strongly agree) to -2 (strongly disagree). A mean score for self-efficacy was composed (Cronbach’s α = 0.68).

Intention to get good academic grades was measured by using the response to one statement as it is done with most studies [10, 48], ‘I intend to get good academic grades’ on a five-point Likert scale from +2 (strongly agree) to -2 (strongly disagree).

#### Academic performance (t3)

Academic achievement was measured using student’s general average of the student’s self-reported performance in all school subjects during a specific semester. The general average is the standard instrument for the assessment of the academic achievement of students in Lebanese schools [49]. All participating schools use a 0–20 scale where the passing grade is 10 out of 20.

### Statistical analysis

All analyses were done using SPSS, version 23 (SPSS Inc., Chicago, Illinois) and statistical significance was set at a p value < 0.05. Data management and cleaning was carried out prior to analysis and revealed that less than 1% of values were missing. Data were presented as means and standard deviations for continuous variables whereas, categorical data were presented as frequencies and percentages.

In order to analyze the first objective, to examine whether parenting style is associated with academic achievement of Lebanese adolescents, bivariate and multivariate analysis were conducted. Bivariate analyses examining the association between academic performance and other continuous variables were carried out using Pearson or Spearman correlation tests. ANOVA test was used for the association between academic achievement and polytomous variables (i.e. parents’ educational level and parenting style). Independent T-test was run to determine if there were differences in academic achievement between males and females, Christians and non- Christians, adolescents living with both parents or not, and adolescents from private schools versus public schools. A multivariate linear regression was carried out to identify which factors were independently associated with academic achievement using the Enter method. Variables which obtained p < 0.1 in the bivariate analysis were entered in the model [50]. Independent variables were introduced by blocks: Model 1 included parenting styles. Model 2 also contained socio-demographic variables. In model 3 the socio-cognitive variables attitude, social norm-teacher and self-efficacy were added. Finally, in model 4, the variable intention was added. Unstandardized Beta coefficients were then reported with their 95% confidence intervals (CI).

**Fig 1:**
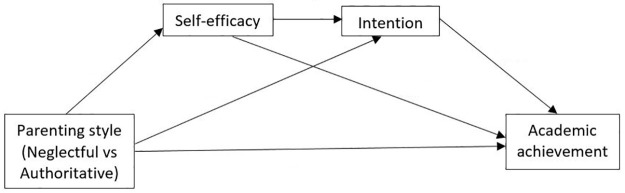
Research framework.

In order to analyze the second objective, to examine whether socio-cognitive factors mediate the effect of parenting style on academic achievement, we conducted the mediation analysis using Hayes’s (2013) SPSS macro PROCESS with 95% bias corrected confidence interval (CI) based on 5000 bootstrap samples [51]. The indirect effect is considered statistically significant if the CI does not include 0. We first started with a simple mediation model using SPSS macro PROCESS (model 4), evaluating one mediator at a time. Among the four socio-cognitive variables (attitude, social norm, self-efficacy and intention) we only obtained significant mediation for self-efficacy and intention. Significant mediators were then entered into a serial mediation model (SPSS macro PROCESS, model 6) to check for significant sequential mediation. We hypothesized a serial mediation model where parenting style influences self-efficacy which in turn influences intention which impacts on academic achievement (Fig 1). In all Hayes models, adjustment was made for the following variables: age, type of school, religion, father and mother education. Confounders were chosen based on the results of the multivariate analysis and the models’ adequacy or goodness of fit. Unstandardized coefficients and standard errors (SE) of the final model were reported alongside their CIs. Direct and indirect effects (DE and IE) were also shown for the final mediation model.

In order to analyze the third objective, to investigate whether adolescent age, gender, school type, religion and parent’s education might moderate the influence of parenting style on academic achievement of Lebanese adolescents, SPSS macro PROCESS model 1 was used. For each potential moderator, four interactions were tested: 1) an interaction with parenting style on academic achievement, 2) an interaction with parenting style on the mediator (socio-cognitive factor), 3) an interaction with the mediator on parenting style, 4) an interaction with the mediator on the sequential mediator. There was no significant interaction with any of the chosen demographic variables.

## Results

### Description of sample

The study sample consisted of 345 adolescents with a mean age of 16.57± 0.76 years. Of the study participants 53.3% were girls and 65.8% attended private school. The majority of adolescents reported an authoritative (31.4%) or authoritarian parenting style (29.3%), followed by permissive (19.9%) and neglectful (19.4%) (Table 1).

**Table 1:** Descriptive characteristics of the sample (N = 345).

Characteristics	Frequency (%)
**Gender**	
Male	161 (46.7)
Female	184 (53.3)
**Age**	
Mean (±SD)	16.57± 0.76
**Type of school**	
Public	118 (34.2)
Private	227 (65.8)
**Religion**	
Christian	302 (87.5)
Non-Christian	43 (12.5)
**Father education**	
Low	29 (8.9)
Medium	149 (45.7)
High	148 (45.4)
**Mother education**	
Low	12 (3.6)
Medium	141 (41.8)
High	184 (54.6)
**Family Structure**	
Live with both parents	317 (91.9)
Other arrangements	28 (8.1)
**Getting good grades is a good help for getting a good job**	
Mean (±SD)	0.69 ± 1.05
**Getting good grades will get me compliment from my parents**	
Mean (±SD)	1.05 ± 0.87
**Getting good grades means that I have to work too hard**	
Mean (±SD)	-0.61 ± 1.02
**Getting good grades means will cause disapproval among my friends**	
Mean (±SD)	1.18 ± 0.94
**Social norm-father**	
Mean (±SD)	1.02 ± 0.91
**Social norm-mother**	
Mean (±SD)	1.18 ±0.77
**Social norm-teacher**	
Mean (±SD)	0.61 ± 0.87
**Self-efficacy Total**	
Mean (±SD)	0.15 ± 0.65
**Intention**	
Mean (±SD)	1.24 ±0.77
**Academic achievement**	
Mean (±SD)	12.89 ± 2.13
Range (over 20)	[6.7;18.5]
**Parenting Style**	
Neglectful	66 (19.4)
Permissive	68 (19.9)
Authoritarian	100 (29.3)
Authoritative	107 (31.4)

### Correlations between academic achievement and parenting styles and socio-demographics: Bivariate analysis

As shown in Table 2, a significantly better academic achievement at t3 was reported for younger adolescents (p = 0.003), those living with both parents (p = 0.035), and adolescents from private schools (p< 0.001). Adolescents whose father (p = 0.001) and mother (p< 0.001) had a high level of education were also significantly more likely to have higher academic achievement at t3. Adolescents with higher reported self-efficacy and with stronger intention at t2 were significantly more likely to have greater academic achievement at t3 (p< 0.001). No significant association of academic achievement with attitude and social norm were found.

**Table 2:** Correlations between academic achievement and parenting styles and socio-demographics: Bivariate associations.

Variables	Academic Achievement Mean ± SD	Test statistic (df)	p
**Gender**		-1.010	0.313 ^a^
• Males	12.77 ± 2.25		
• Females	13.00 ± 2.02		
**Age**	-	-0.163	0.003 ^b^
**Type of school**		-8.589	<0.001 ^a^
• Public	11.65 ± 2.02		
• Private	13.54 ± 1.89		
**Religion**		-2.906	0.004 ^a^
• Christian	13.02 ± 2.11		
• Non-Christian	12.02 ± 2.09		
**Father’s educational level**		6.899	0.001 ^c^
• Low	11.95 ± 1.77		
• Medium	12.62 ± 2.23		
• High	13.33 ±2.07		
**Mother’s educational level**		10.165	<0.001 ^c^
• Low	11.72 ± 1.31		
• Medium	12.42 ± 2.19		
• High	13.36 ±1.99		
**Family structure**		2.119	0.035 ^a^
• Live with both parents	12.96 ± 2.08		
• Other arrangements	12.08 ±2.51		
**Getting good grades is a good help for getting a good job**	-	0.102	0.059 ^b^
**Getting good grades will get me compliment from my parents**	-	-0.015	0.780 ^b^
**Getting good grades means that I have to work too hard**	-	0.066	0.227 ^b^
**Getting good grades means will cause disapproval among my friends**	-	0.020	0.706 ^b^
**My father expects that I get good academic grades**	-	-0.066	0.224 ^b^
**My mother expects that I get good academic grades**	-	-0.005	0.923 ^b^
**My teacher expects that I get good academic grades**	-	0.097	0.074 ^b^
**Self-efficacy**	-	0.272	<0.001 ^d^
**Intention**	-	0.252	<0.001 ^b^
**Parenting style**		2.243	0.083 ^c^
• Neglectful	12.56 ± 2.20		
• Permissive	12.88 ± 2.20		
• Authoritarian	12.69 ± 2.05		
• Authoritative	13.31 ± 2.09		

Notes:

^a^ p-value for the Independent Samples T-test,

^b^ p-value for the Spearman correlation,

^c^ p-value for ANOVA,

^d^ p-value for the Pearson correlation.

### Associations between academic achievement and parenting styles and demographics: Multivariate analysis

In model 1, consisting of parenting styles and academic achievement, the results show that parenting style significantly predicted academic achievement of adolescents six months later. Adolescents whose parents are authoritative were significantly more likely to achieve higher academic achievement six months later compared to adolescents of neglectful (β: -0.87; 95% CI -1.55, -0.19), and authoritarian parents (β: -0.62; 95% CI -1.23, -0.01). Model 2, including model 1 and adding demographic variables, showed that age and type of school were statistically significantly associated with academic achievement at t3. Younger adolescents (p = 0.010) and students from private schools (p< 0.001) were significantly more likely to achieve higher. Parenting remained statistically significantly associated with academic performance at t3. Adolescents of authoritative parents were significantly more likely to have higher academic achievement at six months follow-up compared to adolescents of neglectful (β: -1.07; 95% CI -1.69, -0.45), permissive (β: -0.68; 95% CI -1.29, -0.07) and authoritarian parents (β: -0.83; 95% CI -1.39, -0.27). In model 3, adding the socio-cognitive variables attitude, social norm-teacher and self-efficacy to model 2 resulted in the same significant effect of parenting, age and type of school. It also showed that self-efficacy was positively significantly associated with academic achievement at t3 (p< 0.001). Adolescent with higher self-efficacy had an average grade higher by 0.72 relatively to adolescents with lower self-efficacy. In the last model 4, adding the variable intention to model 3, parenting style, age, school type and self-efficacy remained significant and adding intention as a statistically positively significant predictor of achievement six months later (p<0.001) (Table 3).

**Table 3:** Associations between academic achievement and parenting styles and demographics: Multivariate analysis.

Variables	Model 1			Model 2			Model 3			Model 4		
	β	95% CI	p	β	95% CI	p	β	95% CI	p	β	95% CI	p
**Parenting style**												
• Authoritative	1			1			1			1		
• Neglectful	-0.87	[-1.55, -0.19]	0.012	-1.07	[-1.69, -0.45]	0.001	-0.82	[-1.43, -0.21]	0.008	-0.66	[-1.26, -0.06]	0.032
• Permissive	-0.47	[-1.15, 0.21]	0.173	-0.68	[-1.29, -0.07]	0.029	-0.70	[-1.29, -0.11]	0.020	-0.64	[-1.22, -0.07]	0.029
• Authoritarian	-0.62	[-1.23, -0.01]	0.045	-0.83	[-1.39, -0.27]	0.004	-0.69	[-1.24, -0.16]	0.012	-0.62	[-1.15, -0.09]	0.023
**Age**				-0.38	[-0.67, -0.09]	0.010	-0.35	[-0.63, -0.07]	0.015	-0.31	[-0.59, -0.04]	0.025
**Type of school**												
• Public				1			1			1		
• Private				1.96	[1.42, 2.50]	<0.001	1.86	[1.33, 2.39]	<0.001	1.84	[1.32, 2.36]	<0.001
**Religion**												
• Non-Christian				1			1			1		
• Christian				-0.25	[-0.98, 0.48]	0.502	0.009	[-0.70, 0.72]	0.981	0.09	[-0.60, 0.79]	0.787
**Father’s educational level**												
• Low				1			1			1		
• Moderate				-0.18	[-1.04, 0.67]	0.670	-0.12	[-0.94, 0.71]	0.780	-0.17	[-0.97, 0.64]	0.684
• High				-0.001	[-0.89, 0.88]	0.998	0.07	[-0.78, 0.94]	0.860	-0.004	[-0.85, 0.84]	0.993
**Mother’s educational level**												
• Low				1			1			1		
• Moderate				0.32	[-0.88, 1.52]	0.603	0.08	[-1.07, 1.25]	0.880	-0.08	[-1.22, 1.06]	0.892
• High				0.43	[-0.79, 1.66]	0.487	0.29	[-0.89, 1.49]	0.621	0.19	[-0.98, 1.35]	0.750
**Family structure**												
• Live with both parents				1			1			1		
• Other arrangements				-0.48	[-1.32, 0.35]	0.259	-0.48	[-1.29, 0.32]	0.238	-0.46	[-1.25, 0.33]	0.252
**Getting good grades is a good help for getting a good job**							0.13	[-0.07, 0.33]	0.199	0.09	[-0.10, 0.29]	0.359
**My teacher expects that I get good academic grades**							0.14	[-0.10, 0.38]	0.261	0.09	[-0.14, 0.34]	0.436
**Self-efficacy**							0.72	[0.39, 1.05]	<0.001	0.60	[0.28, 0.93]	<0.001
**Intention**										0.51	[0.24, 0.79]	<0.001

*β* = Unstandardized Coefficient; CI = confidence interval. Dependent variable: Academic Achievement.

**Model 1**: Variables entered: Parenting style (Permissive, authoritarian, neglectful).

Association was significant: p < 0.05

R2 = 0.023

**Model 2**: Variables entered: Variables in Model 1 + Age, Type of school, Religion, Father’s educational level Mother’s educational level, Family structure.

Association was significant: p < 0.05

R2 = 0.257

**Model 3**: Variables entered: Variables in Model 2 + Attitude, Social norm-teacher, Self-efficacy.

Association was significant: p < 0.05

R2 = 0.323

*Model 4*: Variables entered: Variables in Model 3+ Intention

Association was significant: p < 0.05

R2 = 0.352

### Serial mediation analysis

In the serial mediation model, we postulated that our predictor variable parenting style (X) influences academic achievement (Y) six months later via two mediators: self-efficacy (M1) and intention (M2) sequentially. To demonstrate this, parenting style (Y) should be significantly associated with the first mediator (M1) self-efficacy. In turn, self-efficacy (M1) should be significantly associated with the second mediator (M2) intention and finally intention (M2) should be significantly related to academic achievement (Y). Table 4 shows a series of three regression analysis. In the first regression the goal was to examine if our independent variable parenting style (X) measured at t2 is associated with the first mediator (M1) self-efficacy (t2). The results show that parenting style was statistically significantly associated with self-efficacy. Adolescents of neglectful parents were significantly less likely to have high self-efficacy compared to the reference style authoritative (p = 0.022). The second regression, regressed intention (M2) on both self-efficacy (M1) and parenting style (X). The results show that the first mediator M1 (self-efficacy) was statistically significantly associated with the second mediator M2 (intention), with higher efficacy beliefs predicting higher intention (p <0.001). Additionally, parenting was also statistically significantly associated with intention; adolescents of neglectful parents were significantly less likely to report strong intentions (p = 0.006). The last regression analysis regressed academic achievement (Y) on all antecedent variables: intention (M2), self-efficacy (M1) and parenting (X). The goal was to explore if intention (M2) is associated with academic achievement (Y) six months later. The results show that intention (M2) was significantly associated with achievement (Y). Adolescents with greater intention were significantly more likely to have higher achievement (p< 0.001).

**Table 4:** Serial mediation modelling linking parenting style and academic achievement (n = 338).

	**Self-efficacy (Mediator 1)** †				
	Coefficient	SE	P-value	LLCI	ULCI
**Parenting Style**		*Authoritative (ref)*			
• Authoritarian	-0.16	0.09	0.107	-0.35	0.03
• Permissive	0.04	0.11	0.713	-0.17	0.25
• Neglectful	-0.25	0.11	0.022	-0.47	-0.03
	**Intention (Mediator 2)** †				
**Self-efficacy**	0.29	0.06	<0.001	0.03	0.08
**Parenting style**		*Authoritative (ref)*			
• Authoritarian	-0.14	0.11	0.190	-0.36	0.07
• Permissive	-0.13	0.12	0.281	-0.37	0.11
• Neglectful	-0.34	0.12	0.006	-0.58	-0.09
	**Academic achievement (Outcome)** †				
**Intention**	0.53	0.14	<0.001	0.26	0.80
**Self-efficacy**	0.65	0.16	<0.001	0.34	0.96
**Parenting style**	*Authoritative (ref)*				
• Authoritarian	-0.62	0.26	0.020	-1.14	-0.09
• Permissive	-0.64	0.29	0.027	-1.22	-0.07
• Neglectful	-0.73	0.30	0.016	-1.32	-0.13

Serial mediation model taking parenting style as X, academic achievement as Y, and self-efficacy and intention as mediators 1 and 2.

†All models adjusted for age, type of school, religion, father education and mother education. Models using Process Macro model #6 (Hayes, 2013).

The last Table 5, shows the direct effect of parenting style on academic achievement and the three indirect effects: (i) the indirect effect of parenting on achievement through the first mediator self-efficacy (M1), (ii) the indirect effect of parenting on achievement through the second mediator intention (M2) and (iii) the indirect effect of parenting on achievement through the sequence of self-efficacy (M1) and intention (M2). The direct effect of parenting style on the outcome academic achievement is statistically significant (neglectful vs authoritative: DE = -0.73; p = 0.016). In addition, the indirect effect of parenting style (neglectful vs authoritative) on academic performance through the mediator self-efficacy (M1) alone is statistically significant (IE = -0.16; CI = -0.37, -0.002). The second indirect effect of parenting style (neglectful vs authoritative) on academic performance via the second mediator intention (M2) is also statistically significant (IE = -0.18; CI = -0.39, -0.04). Finally, the last indirect effect of parenting style (neglectful vs authoritative) via the sequence of self-efficacy (M1) and intention (M2) leading to academic achievement is statistically significant (IE = -0.04; CI = -0.11, -0.003). Thus, the relationship between parenting style (neglectful vs authoritative) and academic achievement is partially mediated by self-efficacy and intention, sequentially.

**Table 5:** Direct and indirect effects of parenting style on academic achievement.

	**Direct effect**			
	Effect	P-value	LLCI	ULCI
**Parenting Style**	*Authoritative (ref)*			
• Authoritarian	-0.62	0.020	-1.14	-0.09
• Permissive	-0.64	0.027	-1.22	-0.07
• Neglectful	-0.73	0.016	-1.32	-0.13
	**Indirect effects**			
	Parenting style => Self-efficacy => Academic Achievement			
	Effect		Boot LLCI	Boot ULCI
**Parenting Style**	*Authoritative (ref)*			
• Authoritarian	-0.10		-0.25	0.02
• Permissive	0.02		-0.09	0.16
• Neglectful	-0.16		-0.37	-0.002
	Parenting style => Intention => Academic Achievement			
**Parenting Style**	*Authoritative (ref)*			
• Authoritarian	-0.08		-0.24	0.04
• Permissive	-0.07		-0.25	0.05
• Neglectful	-0.18		-0.39	-0.04
	Parenting style => Self-efficacy => Intention => Academic Achievement			
**Parenting Style**	*Authoritative (ref)*			
• Authoritarian	-0.02		-0.07	0.005
• Permissive	0.01		-0.03	0.03
• Neglectful	-0.04		-0.11	-0.003

## Discussion

The first goal of our study was to examine the longitudinal influence of parenting style on academic achievement of adolescents in Lebanon. Secondly, we examined the theoretical mediating role of social cognitive factors in the relationship between parenting style and academic achievement. Thirdly, we examined whether the association between parenting and academic achievement was moderated by age, gender, religion, school type and parents’ education. Our findings indicate that parenting styles was prospectively associated with academic achievement of Lebanese adolescents six month later, this influence was partly mediated by self-efficacy and intention to get good grades. Demographic variables did not moderate the effect of parenting style on academic achievement.

The findings of the present research both support and extend those of previous studies. Our results indicate that authoritative parenting promotes higher academic achievement in adolescents compared to neglectful parenting. The positive influence of authoritative parenting is both direct as well as mediated in part through the effect of self-efficacy and intention. The findings are consistent with other studies showing that self-efficacy act as a mediator between parenting and academic achievement [18, 32, 52]. In addition, this study brings new insight into the literature by suggesting the sequential and causal pathway of self-efficacy and intention in explaining the relation between parenting and achievement which has not been studied before. The association between parenting style and adolescents’ academic achievement was not moderated by age, religion, school type, parents’ education or gender of the adolescents. This is in line with previous research indicating that the influence of parenting on school achievement does not depend on parents’ educational level [53] and that for the most part the influence of parenting styles does not differ across demographic groups [54–56].

Our results from the serial mediation model supports that parenting style significantly influences academic achievement of adolescents both directly and indirectly through proximal cognitive variables. These findings support the dual-process view assumption that the environment-in our case the home environment- may influence behavior directly as well as indirectly through cognitions [24]. Our results show that parenting style influences self-efficacy, which in turn influences intention which subsequently influences academic achievement.

When looking at the direct association of parenting on academic performance, our results show that authoritative parenting had the most positive influence on academic achievement compared to all the other styles. This in line with previous research, even though there are some discrepancies in the findings, the majority of studies report a beneficial effect of authoritative parenting on academic outcome of adolescents [57, 58] and a negative effect of neglectful parenting [19]. Our results indicate that this association is also true for the Lebanese context. Adolescents of neglectful parents were significantly less likely to have high academic achievement than adolescents whose parents are authoritative. Importantly, this association held even after adjusting for socio-demographic and socio-cognitive factors suggesting an independent direct effect of parenting style. The latter implies that parenting programs and interventions aimed at fostering effective and positive parenting is worthwhile considering for improving adolescents’ academic achievement regardless the socio-economical background. Moreover, our findings are also in line with previous studies documenting a positive effect of authoritative parenting on school outcomes among younger children [59, 60]. Hence, even though with age there may be other social influences such as peer relations, parents continue to largely impact their children’s outcomes across adolescence.

Further to the direct effect, our results revealed the indirect mediated effect of parenting on achievement via socio-cognitive factors. Firstly, parenting style was found to significantly directly predict socio-cognitive factors. Adolescents from authoritative parents were more likely to have high academic efficacy beliefs and more likely to have strong intentions towards getting good grades compared to adolescents of neglectful parents. This is in line with previous research showing that authoritative parenting fosters the highest levels of self- efficacy beliefs compared to non-authoritative parenting [18, 61]. This positive influence can be explained by the favorable characteristics of the authoritative style that are assumed to contribute to healthy psychosocial development [20, 62]. Authoritative parents are supportive and responsive towards their children’s need, they foster self-reliance, critical thinking and more positive attitudes towards school [62, 63]. Hence adolescents who describe their parents as being authoritative are more likely to develop positive beliefs about their achievement and be successful in their academic life [64]. Whereas adolescents from neglectful families were found to adopt maladaptive behaviors and task avoidant strategies which inhibit academic achievement [52].

Moreover, socio-cognitive factors were in turn found to be significantly associated with academic achievement. Adolescents with high self-efficacy were more likely to have stronger intention and consequently more likely to perform better compared to their counterparts with lower efficacy beliefs. The latter corroborates previous findings indicating that social cognitive factors are strong determinants of academic success [65, 66]. Adolescents who have strong beliefs in their academic capabilities are more motivated, work harder and with greater persistence even in the face of difficulties and consequently are more likely to set higher goals and develop stronger intention to achieve these goals [67, 68]. On another note, research also suggests that the more students achieve well academically the more they feel confident and the greater their efficacy beliefs [69]. Hence the need for interventions targeting both psychosocial cognitions and academic performance for they are a product of each other. In fact, one of the most influential sources of self-efficacy is mastery experience, past successes enhance efficacy beliefs while experiencing failure lowers it [27]. Interventions that induced successful performances were effective in strengthening adolescents’ self-efficacy and eventually led to academic improvements [70].

In conclusion, our results indicate that authoritative parenting may have a direct positive influence on academic achievement of adolescent as well as indirect influence through psychosocial competencies, namely higher self-efficacy, which influence intention which most likely translate into direct action to get good academic achievement. Our findings suggest the need for interventions aiming at encouraging positive parenting styles and promoting positive relationship with adolescent for enhancing efficacy beliefs which may subsequently result in improved academic achievement. Parenting interventions are considered a promising approach and the recommended strategy for enhancing parent-child relationship [71, 72]. Several parenting training programs exist today such as the Triple P-Positive Parenting Program [73] and Parent Management Training [74]. Those programs are aimed at empowering parents and supporting them to adopt positive parenting techniques, reduce parental stress and strengthen parent-child relationship [75]. Consequently, good parent-child relationship and competent parenting may positively influence several child emotional and behavioral outcomes [76, 77] among which academic outcomes [78].

It is important to note that the association between parenting and achievement was partly mediated by cognitions which implies the presence of other potential mediators, such as context-specific parenting practices which have been found to be significantly linked to academic achievement [15, 79]. Positive parenting practices aimed at improving school success such as parental involvement, have been suggested to positively influence academic outcome when occurring under a positive parenting style such as an authoritative home environment [80]. Therefore, specific parenting practices may mediate the influence of general parenting style on academic achievement and should be included when examining the relation of general parenting to student’s achievement. Another important factor to consider is motivation, prior work has found that motivation prospectively influence academic achievement [81] and is influenced by parenting style [82]. Hence the importance of examining the moderating or mediating role of motivation in the relation of parenting to intention and academic achievement in future research.

### Strength & limitations

To the best of our knowledge this is the first study to prospectively examine the direct and indirect influence of parenting style on academic achievement of Lebanese adolescents. The current research presents new evidence on parenting socialization in the Lebanese culture and highlights the importance of the home environment in influencing Lebanese adolescents school outcomes. In addition, testing both the direct and indirect effect of parenting styles brings new insight in understanding the potential mechanisms by which the family environment influences adolescent academic achievement. An important contribution of this study is the sequential pathway of self-efficacy and intention, as examined by the serial mediation model, that provides a new explanation into the direct and indirect link between parenting style and academic achievement through the mediator effect of self-efficacy and intention. An additional strength of this study is the methodological approach used to test mediation using Hayes’s macro PROCESS based on bootstrapping which bypasses the problem of distributional assumption and provides more power in detecting indirect effect even in small samples [83].

Nonetheless, a number of limitations of this study need attention. First, due to the fact that grades were self-reported, common response bias cannot be ruled out. However, previous research indicate that self-reported grades are accurate indicators of actual grades and are comparable to academic transcript [84, 85]. In addition, our sample was taken from two areas in Lebanon, Beirut the Capital city and Mount Lebanon are. These two regions concentrate the majority of the Lebanese population, including approximately half of the Lebanese students, and are representative of the various religious and socio-demographic societies in Lebanon. However, the sample is not at a national level and, consequently, the findings cannot be generalized to the whole adolescent population in Lebanon. Furthermore, our study examines parenting style in relation to both parents rather than individually and thus we were unable to explore the ways in which mothers and fathers differed in their parenting styles. Future studies examining the parenting styles of the mother and father separately are recommended as the cultural socializing pattern might differ. Finally, our research focused on the more global aspect of parental behavior that is parenting style and did not include behavior-specific parenting practices aimed at directly promoting school achievement. The inclusion of parenting practices is recommended in future research as domain-specific parenting behaviors (such as monitoring school activities) might produce larger effects than general parenting. In addition, examining both parenting styles and parenting practices will enable us to examine how general parenting may moderate the influence of specific parenting practices on academic achievement.

## Conclusion

Our findings extend previous evidence on the positive influence of authoritative parenting on academic achievement both directly and indirectly through the mediating effect of self-efficacy and intention and highlight the importance of promoting this style for enhancing academic performances as well as psychosocial well-being among Lebanese adolescents. Future research may incorporate, motivation, peer relations and parenting practices as additional potential mediators as they have been found to be influenced by parenting style and significantly relate to school achievement.

## Supporting information

S1 Dataset (SAV)

S1 File Survey items. (PDF)

S1 Fig Associations between academic achievement and parenting styles and demographics: Multivariate analysis by block. (TIFF)
