# Supplementary material for: Authoritative parenting stimulates academic achievement, also partly via self-efficacy and intention towards getting good grades
Source: PLoS One. 2022 Mar 30;17(3):e0265595. doi: 10.1371/journal.pone.0265595 (PMC8967044; doi:10.1371/journal.pone.0265595)
Supplement: S1 File — (PDF) [file pone.0265595.s002.pdf]

**Socio-demographics**			
	Questions	Answer	
1.1	Gender	1=Male<br>2=Female	
1.2	How old are you?	1=15 years old<br>2=16 years old<br>3=17 years old<br>4=18 years old	
1.10	What is the highest level of education that your father has completed?	1=Never went to school<br>2=Completed primary school<br>3=Completed complementary school<br>4=Completed secondary school<br>5=Technical school<br>6=University<br>7=I don't know	1=low: never went to school & primary school<br>2=medium: complementary & secondary school<br>3=high: technical school & university
1.11	What is the highest level of education that your mother has completed?	1=Never went to school<br>2=Completed primary school<br>3=Completed complementary school<br>4=Completed secondary school<br>5=Technical school<br>6=University<br>7=I don't know	1=low: never went to school & primary school<br>2=medium: complementary & secondary school<br>3=high: technical school & university
1.14	Who lives with you in your home?<br>Family structure	1=Both parents<br>2=Mother and not father<br>3=Father and not mother<br>Other, specify _________________	1=living with both parents<br>2=other arrangements
1.15	What is your religion?	1=Christian<br>2=Muslim<br>3=Atheist<br>If other specify:_______________	

	**Socio-cognitive factors**	
1.1	Getting good academic grades is a good help for getting a good job	Attitude Pro
1.2	Getting good academic grades will get me compliment from my parents	
1.3	Getting good academic grades means that I have to work too hard	Attitude Con
1.4	Getting good academic grades will cause disapproval among my friends	
1.5	My father expects me to get good academic grades	Social norms
.16	My mother expects me to get good academic grades	
1.8	My teacher expects me to get good academic grades	
1.9	I find it very easy to get good academic grades	Self-efficacy
1.10	I find it easy to concentrate at school for getting good academic grades	
1.11	I find it easy to master the skills that are taught in class this year	
1.12	I find it easy to concentrate on school work when I am at home	
1.13	I find it easy to finish all my school work	
1.14	I intend to get good academic grades	Intention

Responses: 1 = strongly disagree, 2= Disagree, 3= Neither agree nor disagree, 4= Agree 5 = strongly agree

We asked other teenagers what their parents are like. Listed below is what these other teenagers said. Please tick the box that shows how much the following statements are like your parents.

	Not like them	Sort of like them	A lot like them	Just like them
They are always telling me what to do.	□	□	□	□
They make rules without asking what I think.	□	□	□	□
They make me feel better when I am upset.	□	□	□	□
They are too busy to talk to me	□	□	□	□
They listen to what I have to say.	□	□	□	□
They like me just the way I am.	□	□	□	□
They tell me when I do a good job on things.	□	□	□	□
They want to hear about my problems.	□	□	□	□
They are pleased with how I behave.	□	□	□	□
They have rules that I must follow.	□	□	□	□
They tell me times when I must come home.	□	□	□	□
They make sure I tell her where I am going.	□	□	□	□
They make sure I go to bed on time.	□	□	□	□
They ask me what I do with friends.	□	□	□	□
They know where I am after school.	□	□	□	□
They check to see if I do my homework.	□	□	□	□
