# Supplementary figures and images for: Authoritative parenting stimulates academic achievement, also partly via self-efficacy and intention towards getting good grades
Source: PLoS One. 2022 Mar 30;17(3):e0265595. doi: 10.1371/journal.pone.0265595 (PMC8967044; doi:10.1371/journal.pone.0265595)

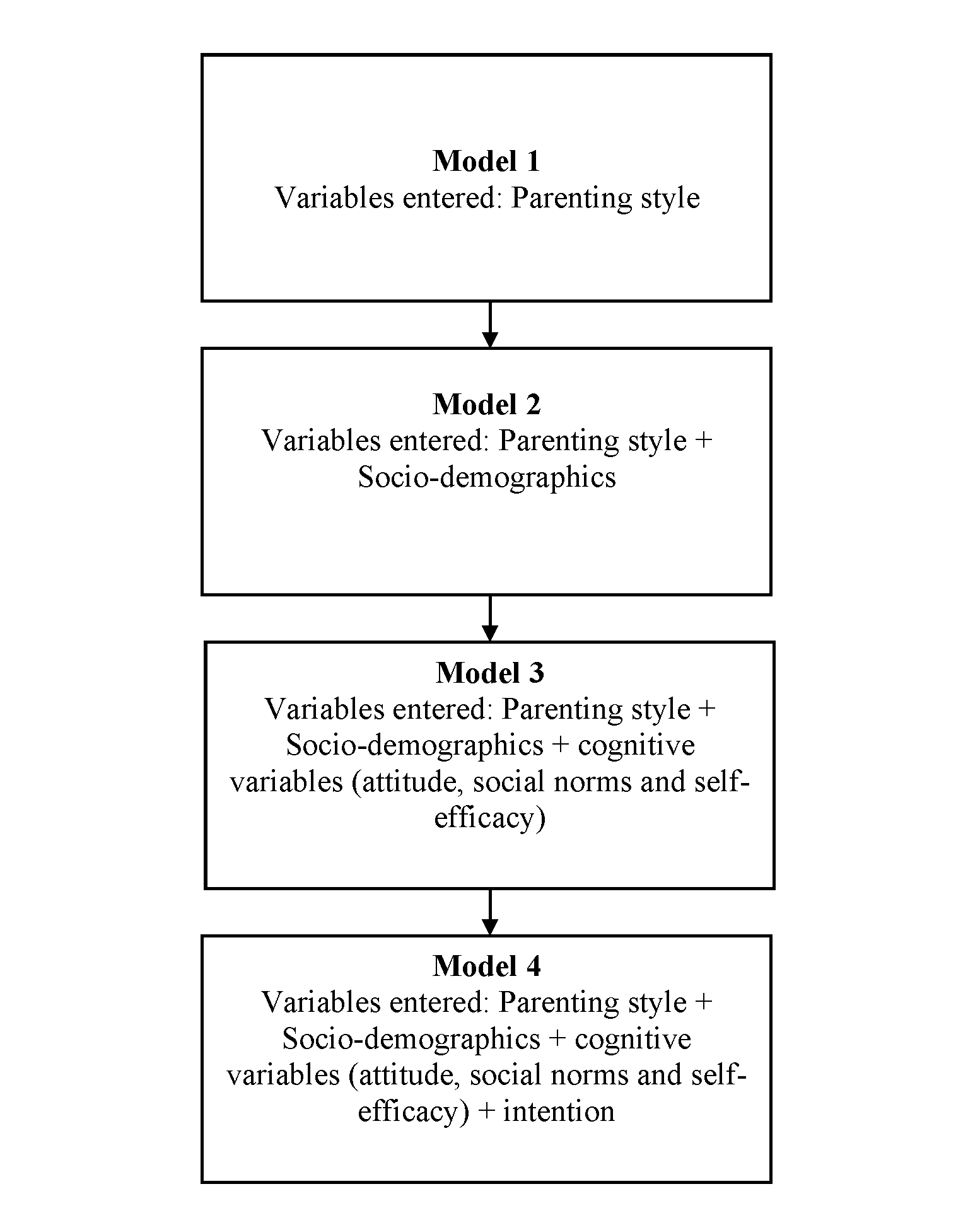

Supplement: S1 Fig — (TIFF) [file pone.0265595.s003.tiff]
